# The FAM83 family of proteins: from pseudo-PLDs to anchors for CK1 isoforms

**DOI:** 10.1042/BST20160277

**Published:** 2018-06-05

**Authors:** Polyxeni Bozatzi, Gopal P. Sapkota

**Affiliations:** MRC Protein Phosphorylation and Ubiquitylation Unit, School of Life Sciences, University of Dundee, Dow Street, Dundee DD1 5EH, U.K.

**Keywords:** cancer, casein kinase, CK1, FAM83, PLD, Wnt proteins

## Abstract

The eight members of the FAM83 (FAMily with sequence similarity 83) family of poorly characterised proteins are only present in vertebrates and are defined by the presence of the conserved DUF1669 domain of unknown function at their N-termini. The DUF1669 domain consists of a conserved phospholipase D (PLD)-like catalytic motif. However, the FAM83 proteins display no PLD catalytic (PLDc) activity, and the pseudo-PLDc motif present in each FAM83 member lacks the crucial elements of the native PLDc motif. In the absence of catalytic activity, it is likely that the DUF1669 domain has evolved to espouse novel function(s) in biology. Recent studies have indicated that the DUF1669 domain mediates the interaction with different isoforms of the CK1 (casein kinase 1) family of Ser/Thr protein kinases. In turn, different FAM83 proteins, which exhibit unique amino acid sequences outside the DUF1669 domain, deliver CK1 isoforms to unique subcellular compartments. One of the first protein kinases to be discovered, the CK1 isoforms are thought to be constitutively active and are known to control a plethora of biological processes. Yet, their regulation of kinase activity, substrate selectivity and subcellular localisation has remained a mystery. The emerging evidence now supports a central role for the DUF1669 domain, and the FAM83 proteins, in the regulation of CK1 biology.

## Introduction

**Figure 1.**
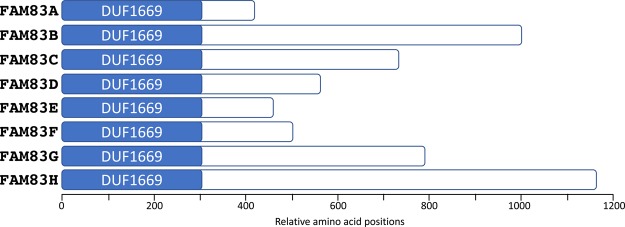
The FAM83 family of proteins. Schematic representation of the eight members (A–H) of the FAM83 proteins indicating the N-terminal conserved domain of unknown function DUF1669. The C-terminus of each FAM83 member contains unique amino acid sequences that do not offer many functional clues. The positions of the amino acids relative to the DUF1669 domain are indicated for each member.

The FAM83 (FAMily with sequence similarity 83) family of proteins consists of eight members (A–H), which have been classified based on the sequence similarity of a conserved domain of unknown function, namely the DUF1669 domain (Pfam PF07894). The conserved globular DUF1669 domain lies at the very N-termini of the FAM83 members (Figure 1). Outside the DUF1669 domain, there is no detectable sequence similarity among the family members and the whole C-terminal parts of every FAM83 member are predicted to consist of non-globular disordered regions (Figure 1) [1–3]. Domains, by definition, are functional and/or structural units of a protein [4]. Thus, grouping proteins together based on the presence of a conserved domain can provide a useful starting point to build testable hypotheses, especially when there is a lack of any functional information. Therefore, establishing the biochemical role(s) of the DUF1669 domain is pertinent to uncovering the biological roles of the FAM83 family of proteins. The genes encoding the FAM83 family of proteins are present only in vertebrates.

## Are FAM83 proteins pseudo-PLDs?

**Figure 2.**
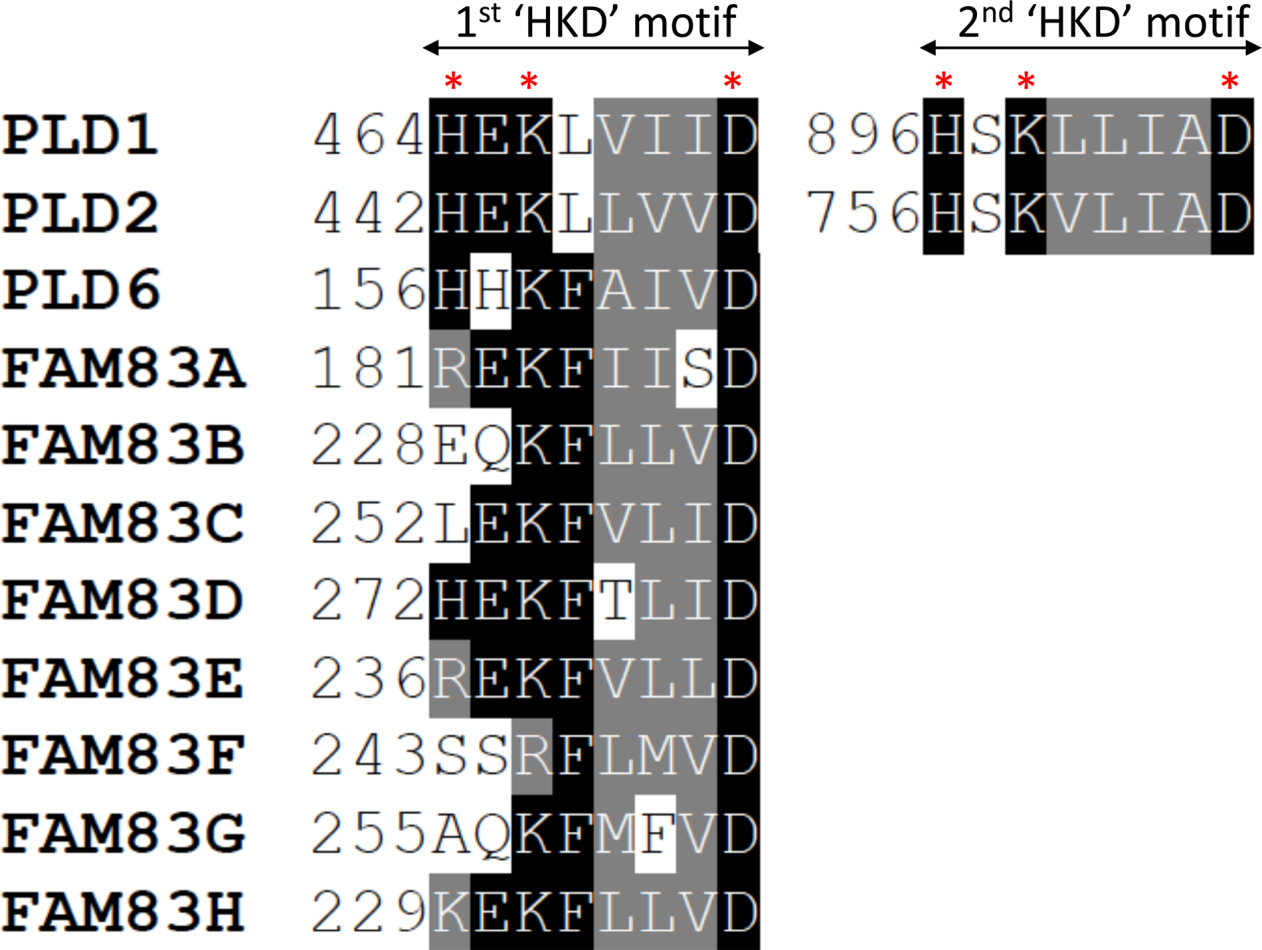
FAM83 proteins each contain one pseudo-PLDc motif. The **‘**HKD’ motifs, which define the catalytic core of the PLD enzymes, from human PLD1 and PLD2 (which contain two HKD motifs) and PLD6 (which contains one motif) are aligned with those from the FAM83 members using the Clustal Omega and BoxShade server. The first histidine (H) residue as well as identical residues conserved in more than 50% of the sequences are boxed in black and similar residues are boxed in grey. The positions of the HKD residues within the motifs are indicated by asterisks. All FAM83 proteins, except FAM83D, lack the first histidine and are therefore termed *pseudo-PLDs*.

According to annotation databases (UniProt and SMART), the DUF1669 domain of FAM83 proteins displays a single short stretch of amino acids that resembles the catalytic motif of phospholipase D (PLD) enzymes. The conventional PLD catalytic (PLDc) motif comprises the conserved consensus sequence His-x-Lys-x-x-x-x-Asp/Glu (also referred to as HKD motif), where x denotes any amino acid (Figure 2) [5,6]. All human PLDs, except PLD6, have two HKD motifs within a single polypeptide, and both are critical for their enzymatic activity [5,7]. Notably, PLD6 is phylogenetically closer to the bacterial endonuclease Nuc, which also contains a single HKD motif [7]. However, the crystal structure of Nuc showed that it dimerises to form a functional complex [8]. Although dimerisation between either the two intrinsic HKD motifs of a single PLD enzyme or two HKD motifs from distinct PLDs have been suggested for active complexes [9], these have not been confirmed by structural studies. PLDs hydrolyse the most abundant membrane lipid, phosphatidylcholine, to choline and phosphatidic acid [5]. Phosphatidic acid is rapidly converted into diacylglycerol by phosphatidic phosphatase, which then activates protein kinase C isoforms [5,10]. PLDs are involved in phospholipid metabolism, intracellular signal transduction and vesicle trafficking [11]. There are six PLD isoforms (PLD1–6) in humans. PLD1 and PLD2 share ∼50% sequence similarity and are ubiquitously expressed. PLD3 and PLD4 are transmembrane proteins anchored to the endoplasmic reticulum, with the HKD domains exposed to the lumen. PLD5 is catalytically inactive, as it lacks the HKD motifs. PLD6 is localised to the mitochondria and has a single HKD motif [12]. From structural and biochemical studies of many PLDs, the enzymatic mode of action of the PLD superfamily is well understood [5]. The ‘histidine’ of the conserved ‘HKD’ motif mediates a nucleophilic attack on the phosphate group of the lipid substrate (e.g. phosphatidylcholine) and yields a covalent intermediate. Subsequently, a water molecule or a primary alcohol completes the hydrolysis or transphosphatidylation, respectively [5]. Therefore, the ‘histidine’ residue of the ‘HKD’ motif is critical for catalysis and is often mutated to ‘alanine’ in PLDs in order to abolish their catalytic activity.

In the case of the DUF1669 domain of the FAM83 proteins, the assignment of the putative HKD motif is rather ambiguous: (i) in all FAM83 members, except FAM83D, the ‘histidine’ of the ‘HKD’ motif is substituted with a different residue, and the ‘lysine’ residue in FAM83F is substituted with an ‘arginine’ residue, suggesting an atypical motif that we refer to as ‘pseudo-HKD’ motif (Figure 2); (ii) only one pseudo-HKD motif exists in each FAM83 protein, instead of two seen in most PLDs; (iii) outside the pseudo-HKD motif, there is very little sequence similarity between the DUF1669 domain and PLDs and (iv) to date, no direct PLDc activity has been attributed to any FAM83 protein. We have been unable to detect PLDc activity with any human FAM83 member expressed in bacteria, including FAM83D, which has an intact HKD motif (unpublished findings). However, preliminary structural analysis from two FAM83 members suggest that the positioning and architecture of the pseudo-HKD motifs appears to be similar to that seen in PLD structures [13,14]. Therefore, based on the presence of the pseudo-HKD motif that potentially conforms to the HKD-like fold in PLDs, the FAM83 proteins could be classified as pseudo-PLD enzymes. A comprehensive analysis of the structures of the DUF1669 domain and PLDs is needed to ascertain how and why the FAM83 proteins have lost the PLDc activity. Such analyses will provide insights into the evolution of the FAM83 family of pseudoenzymes. Given that the histidine residue of the HKD motif appears to be the most targeted substitution in FAM83 proteins (except FAM83D), it might be interesting to test whether restoring this histidine back could reinstate the PLDc activity of FAM83 proteins. In some pseudophosphatases, a simple back-mutation (typically reinstating the catalytic Cys residues) is often known to convert them ‘back’ to enzymatically active phosphatases [15].

Pseudoenzymes can be defined by an absence of detectable catalytic activity. They are thought to have evolved from gene duplication events, which remove the selective pressure on the catalytic activity from additional copies and allow them to mutate and adopt new functions [16–18]. Although the full extent of the biological roles of pseudoenzymes is currently unknown, evidence from many studies reveals that pseudoenzymes can act as allosteric modulators of their ancestral enzymes, competitors for binding substrates or regulators, molecular switches and hubs for assembling signalling complexes [16,17]. Currently, we have limited evolutionary, structural and biochemical insights into the FAM83 family proteins to confidently ascertain whether they can be classified as conventional pseudo-PLDs. In this context, however, it is interesting to note that a study reported that FAM83B overexpression in the human mammary epithelial HME1 cell line resulted in increased PLD1 activity, although this was attributed to an indirect activation through activation of the epidermal growth factor receptor (EGFR) [19].

In the absence of concrete functional evidence from the amino acid composition of the FAM83 proteins, including the DUF1669 domain, clues to their potential roles have mainly come from studies looking at different, often unrelated cellular and physiological processes. The findings from some of these studies on the roles of FAM83 proteins are summarised below. These studies point to the extensive range of cellular processes that these members are implicated to influence. It is not surprising then that the diversity of the proposed biological roles for different FAM83 proteins might turn out to be the biggest clue that unites the FAM83 proteins as crucial regulators of the pleiotropic CK1 (casein kinase 1) isoforms.

## The role of FAM83A and FAM83B in chemoresistance and oncogenesis

FAM83A was uncovered as a mediator of tyrosine kinase inhibitor resistance from a phenotypic screen looking for proteins whose overexpression was capable of reversing the growth inhibition of an isogenic clone (T4-2) of the malignant HMT3522 human breast cancer cell line series caused by the small-molecule EGFR inhibitor AG1478 [20]. It was shown that the overexpression of FAM83A in these cells increased their proliferation and invasion rates, while its depletion reversed these phenotypes and rendered these cells sensitive to tyrosine kinase inhibitors [20]. It was proposed that FAM83A interacts with and promotes the phosphorylation of c-RAF and PI3K p85, leading to activation of the mitogen-activated protein kinase (MAPK) pathway downstream of EGFR [20]. Notably, another pseudo-enzyme, the kinase suppressor of RAS (KSR) has been reported to form complexes with MEK and Raf, thereby acting as a scaffold to promote phosphorylation of MEK by Raf [21]. The potential involvement of different pseudoenzymes in MAPK signalling highlights the complexity and adaptability of signalling in eukaryotes. FAM83B was unearthed as a mediator of oncogenic transformation from a screen aimed at identifying proteins whose overexpression could substitute for Ras-mediated transformation of immortalised human mammary epithelial cells (HMECs) [22]. FAM83B was also found to interact with RAS and cRAF and mediate the activation of the MAPK cascade downstream. It was proposed that FAM83B disrupted the association between cRAF and 14-3-3, causing increased cRAF membrane localisation [23]. RNAi-mediated depletion of FAM83B was shown to inhibit the proliferation of RAS-transformed HMECs and attenuate MAPK signalling [23]. Subsequently, it was also shown that overexpression of FAM83B activates the PI3K/AKT pathway and bestows reduced sensitivity to small-molecule inhibitors of PI3K, AKT and mTOR [23]. It was further proposed that FAM83B-mediated hyperactivation of EGFR causes activation of PLD1, which appears to be essential for transformation of the cells [19]. Similarly, overexpression of FAM83C, FAM83D and FAM83E also caused transformation of HMECs, and the overexpression of the DUF1669 domain alone was sufficient for promoting transformation [24]. Some recent reports, by primarily citing publicly available gene or protein expression and mutation databases, have suggested FAM83 proteins as potential biomarkers of some cancers [25], and that expression of some FAM83 members could inform therapeutic strategies [26], and predict survival outcomes in patients [2]. Similarly, cBioPortal for Cancer Genomics and COSMIC (Catalogue of Somatic Mutations in Cancer) databases have catalogued many mutations spread throughout the sequence of different FAM83 proteins in different cancers, although none have yet been validated experimentally and it is likely that many of these mutations are passenger mutations.

## The role of FAM83D in cell cycle progression and proliferation

FAM83D (also known as CHICA) was identified as an interactor of the chromokinesin KID and was found to be involved in localising KID to the spindle during mitosis [27]. It has also been shown to associate with other spindle-localised proteins, namely dynein light chain 1 (DYNLL1) and HMMR, and govern the metaphase plate organisation and spindle orientation [28,29]. RNAi-mediated depletion of FAM83D caused misalignment of chromosomes, shorter spindles and delay in mitosis [27,28]. In many cancers, FAM83D mRNA expression has been suggested to offer prognostic value, with elevated FAM83D expression significantly predicting lower survival rates [29–32]. It has also been reported that FAM83D promotes cell proliferation in hepatocellular carcinoma by either promoting the MAPK signalling pathway [33,34] or suppressing the tumour-suppressor gene FBXW7 [35].

## The role of FAM83G in cell signalling, morphology and disease

From a proteomic screen, we reported the identification of FAM83G (also known as PAWS1; protein associated with SMAD1) as an interactor of the bone morphogenetic protein (BMP) pathway mediator SMAD1 [3]. PAWS1 is involved in modulating the non-canonical (i.e. SMAD4-independent) BMP signalling and is also a substrate for type I BMP receptor kinases [3]. We found that PAWS1 regulated the transcription of many genes independent of BMP signalling, suggesting roles beyond BMP signalling [3]. PAWS1 deficiency causes severe defects in F-actin organisation and distribution, as well as lamellipodial organisation, resulting in impaired cell migration. The dynamic association of PAWS1 with the actin/cytoskeletal regulator CD2AP at lamellae appears to be essential for PAWS1 to control actin organisation and cellular migration [36]. Interestingly, deficiency of CD2AP phenocopies the actin and cell migration defects observed when cells are depleted of PAWS1 [36]. Recently, we showed that PAWS1 overexpression in *Xenopus* embryos activates Wnt signalling and causes complete axis duplication. Wnt signalling is diminished in PAWS1-knockout U2OS osteosarcoma cells, suggesting a critical role for PAWS1 in mediating Wnt signalling [37]. Phenotype-driven transgenic mice displaying spontaneous wooly hair phenotype were mapped to homozygous mutations on the *FAM83G* gene [38]. Two autosomal recessive point mutations on the *FAM83G* gene in dogs (R52P) and humans (A34E) have been reported to cause hereditary palmoplantar (footpad) hyperkeratosis syndrome, which is characterlocalised by thickening of footpads, epidermal hyperplasia and abnormal hair morphology [39–41]. Given the fundamental roles of Wnt signalling in regulating skin and hair development [42–44], these phenotypes may be associated with abnormal Wnt signalling which we have shown is regulated by PAWS1 [37].

## The emerging roles of FAM83H in Amelogenesis imperfecta, keratin cytoskeleton and cancer

Autosomal dominant mutations in FAM83H, many of which are predicted to encode truncated fragments of FAM83H protein, have been reported in patients with hereditary hypocalcified Amelogenesis imperfecta (AI) [45–48]. AI is a genetic disease characterised by defects in the enamel, resulting in a range of tooth disorders [46]. The precise molecular mechanisms through which FAM83H mutations result in AI remain unclear. However, the observations of normal thickness, density and morphology of enamel in FAM83H-knockout mice suggest that the pathogenicity of FAM83H mutations is most likely due to the acquisition of a novel function by the truncated FAM83H mutants, perhaps through disruption of the subcellular distribution of these fragments and associated proteins [49–51]. FAM83H is thought to regulate the keratin cytoskeleton through its interaction and co-localisation with keratins and CK1α [52]. Both overexpression and knockdown of FAM83H as well as chemical inhibition of CK1α were reported to result in the disorganised keratin cytoskeleton [52]. In ameloblast cells, in addition to keratin filaments, FAM83H and CK1α were shown to localise to desmosomes, which provide intercellular junctions [49]. In these cells, it was shown that the overexpression of truncated pathogenic AI mutants of FAM83H resulted in disorganised keratin cytoskeleton [49]. Elevated expression of FAM83H has been associated with colorectal cancer [53] and hepatocellular carcinoma [54]. Recently, FAM83H and CK1 isoforms were shown to co-localise in nuclear speckles with a DNA-binding protein, SON [53].

## The roles of FAM83C, FAM83E and FAM83F remain to be defined

Other than the oncogenic potential discussed above, no biochemical or biological roles of FAM83C, FAM83E and FAM83F have yet been reported. Two microRNAs miR-143 [55] and miR-455-3p [56] have been reported to suppress oesophageal squamous cell carcinomas by targeting the suppression of FAM83F mRNA expression.

## A proteomic approach identifies CK1 isoforms as interactors of all FAM83 proteins

In search for possible clues that could shed light on potential biochemical roles of the conserved DUF1669 domain, we undertook a comprehensive proteomic approach to identify protein interactors of all the FAM83 proteins [57]. Reflecting the diversity in amino acid composition that the FAM83 members display outside the conserved DUF1669 domain, many unique interactors were identified for each member, including SMAD1 for PAWS1 and HMMR and DYNLL1 for FAM83D [3,28]. Excitingly, however, we found that all FAM83 proteins interacted with at least one or more of α, α-like, δ and ε isoforms of the of CK1 family of Ser/Thr protein kinases. All FAM83 members interact with CK1α and α-like isoforms, but only half of them (A, B, E and H) also interact with CK1δ and ε isoforms [57]. We also established that the DUF1669 domain of FAM83 proteins was essential and sufficient in mediating the interaction with CK1 isoforms [57] and that the interaction between the DUF1669 domain and CK1 isoforms appears to be direct [57]. Furthermore, by swapping the DUF1669 domain from one FAM83 member to the other, we were able to alter the ability of the FAM83 members to bind to specific CK1 isoforms [57]. There appears to be a difference in affinity with which each FAM83 member interacts with different CK1 isoforms, suggesting that there could be a possible regulation of the interaction [57]. Interestingly, a study reporting an unbiased analysis of historic interlaboratory proteomic data on 32 protein kinase interactors also revealed several FAM83 proteins as interactors of CK1α and CK1ε isoforms [58].

**Figure 3.**
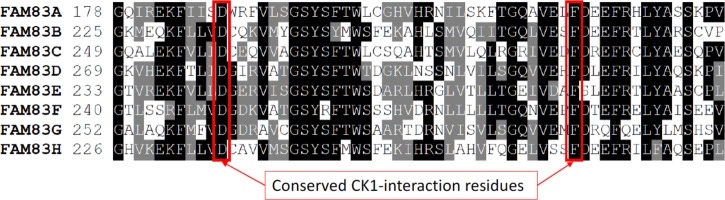
Alignment of the region in FAM83 DUF1669 domain that contains CK1-interacting residues. The indicated regions of the DUF1669 domains of the FAM83 proteins are aligned using the Clustal Omega and BoxShade server. Identical residues conserved in more than 50% of the sequences are boxed in black and similar residues are boxed in grey. The conserved Asp (D) and Phe (F) residues, which abolish CK1 interaction when individually mutated to Ala (A), are indicated. Interestingly, the same Asp (D) residue is part of the HKD motif in FAM83 proteins.

It was proposed that FAM83H interacts with CK1 through the F-x-x-x-F motif, where x denotes any amino acid [52]. This motif was previously identified as a CK1-binding motif in NFAT1 and PER1/2 proteins, where mutation of either phenylalanine residue abolished CK1 interactions [59]. Although FAM83H has four F-x-x-x-F motifs within the DUF1669 domain, mutation of only a single phenylalanine to alanine (F274A) was shown to diminish its association with CK1, whereas the F251A mutation from another F-x-x-x-F motif did not [52]. F274 of FAM83H is uniquely positioned, so that it is flanked by two other phenylalanine residues (F270 and F278) at the −4 and +4 positions, making it part of two consecutive F-x-x-x-F motifs (Figure 3). Interestingly, the F-x-x-x-F motif composed of F270 and F274 in FAM83H is conserved within the DUF1669 domains of all FAM83 proteins, while F278 is not conserved. Rather surprisingly, when we mutated the equivalent of the conserved FAM83H–F274 residue in FAM83G/PAWS1 (F300A), the ability of PAWS1 to interact with CK1, activate Wnt signalling and cause axis-duplication phenotype in *Xenopus* embryos was unaffected [37]. Instead, we determined that mutating the first phenylalanine residue (i.e. the equivalent of FAM83H–F270) from the conserved F-x-x-x-F motif in FAM83E–H to alanine substantially inhibited their interaction with CK1 isoforms (Figure 3) [57]. These observations suggested that the existence of the F-x-x-x-F motif in FAM83 proteins was not absolutely necessary for mediating the CK1 association [37]. Instead, we postulated that there could be a structural moiety within the DUF1669 domain that could be responsible for the tight association between the DUF1669 domain and CK1 isoforms. Consistent with this notion, we performed a mutational scan of conserved residues within the DUF1669 domain of FAM83G and unearthed another mutation, D262A, that also abolished the interaction with CK1α [37]. This aspartate residue, which is the same one that forms the pseudo-HKD motif, is conserved in all FAM83 proteins and when mutated to alanine from FAM83E–H, the interaction with CK1 isoforms was also abolished (Figure 3) [37,57].

## CK1: a protein kinase family longing for regulation!

**Figure 4.**
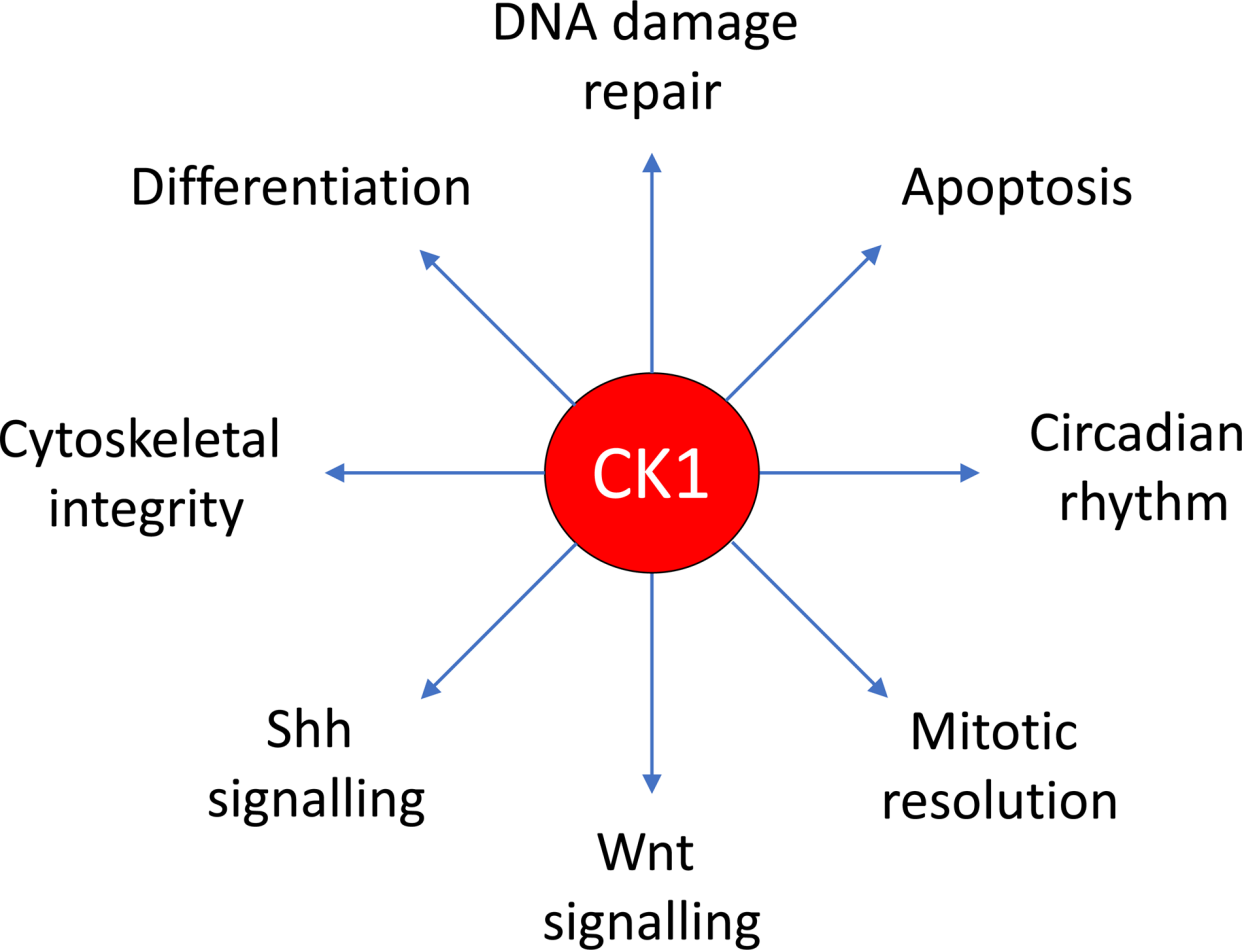
CK1 isoforms control many cellular processes. A schematic representation of the many biological processes that CK1 isoforms are known to regulate. How the activity of CK1 isoforms is regulated for them to influence so many cellular processes remains a mystery.

The CK1 isoforms constitute one of the first serine/threonine protein kinase families to be identified [60]. The family consists of a group of evolutionarily conserved proteins that are ubiquitously expressed in eukaryotic cells and thought to be constitutively active kinases [61–63]. In humans, there are seven CK1 isoforms encoded by different CSNK1 genes (α, α-like, γ1, γ2, γ3, δ and ε), which contain a conserved N-terminal kinase domain and a small, divergent C-terminus. Within CK1 isoforms, the kinase domains of CK1α and α-like, CK1δ and ε, and CK1 γ1–3 display higher overall homologies, while the three CK1γ isoforms diverge substantially from the rest of the family [64]. Although not essential, CK1 isoforms show a preference for an acidic residue or a phospho-serine/threonine residue at *n*−3 (where *n* denotes the CK1 phosphorylation residue). CK1 isoforms are known to localise to many different subcellular compartments and phosphorylate many target proteins, including transcription factors, adhesion proteins, cytoskeletal components, membrane receptors, ribosomal proteins, vesicle-associated proteins, metabolic enzymes and many other signalling molecules, in response to different signalling cues (Figure 4) [62,65]. However, the regulation of their activity, substrate specificity, subcellular localisation and stability in cells remains poorly defined. In view of the vast range of substrates that CK1 isoforms phosphorylate, it is not surprising that CK1 isoforms are involved in controlling many cellular processes, including Wnt and Shh signalling, circadian rhythm, cell cycle progression, cell proliferation, DNA damage repair and apoptosis [61,62,64,65]. Consequently, aberrant CK1 activity is thus associated with many diseases, including cancer [64].

## FAM83 proteins serve to localise CK1 isoforms to distinct subcellular compartments

**Figure 5.**
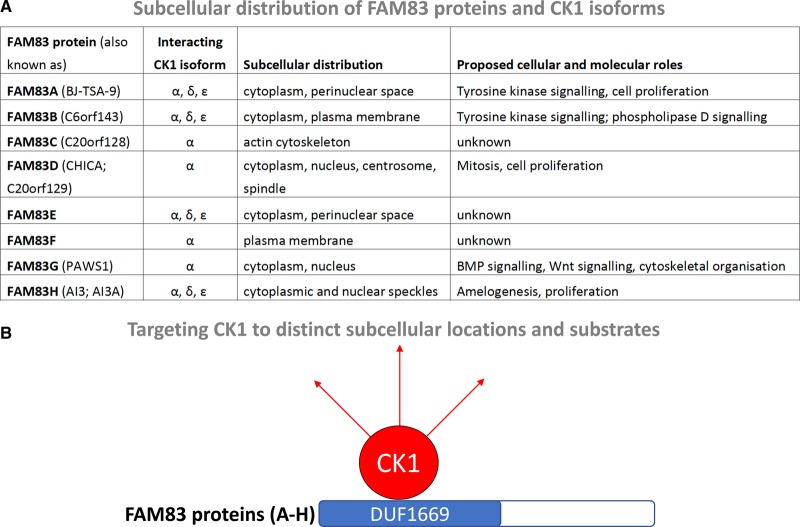
The FAM83 proteins act to target CK1 isoforms to distinct subcellular compartments and, potentially, substrates. (**A**) FAM83 proteins and different CK1 isoforms colocalise in distinct subcellular compartments. (**B**) It is hypothesised that the FAM83 proteins target different CK1 isoforms to distinct subcellular compartments and substrates in response to different signals. The DUF1669 domain of FAM83 proteins serves as a CK1-binding domain.

The observations that FAM83 proteins interact robustly with different CK1 isoforms through the conserved DUF1669 domain raised a tantalising possibility that the FAM83 family could act as key regulators of CK1 isoforms. When we analysed the subcellular distribution of FAM83 members in cells, we observed that different members displayed distinct localisation patterns. FAM83A displayed pan-cellular staining, with strong perinuclear punctate structures. FAM83B was localised to the plasma membrane and the cytoplasm. FAM83C showed localisation along cortical actin stress fibres. FAM83D displayed cytoplasmic staining, with some punctate staining in the nucleus, and it has been reported to localise to the spindle apparatus during mitosis [27,28]. FAM83E exhibited cytoplasmic and strong perinuclear localisation. FAM83F was detected at the plasma membrane. FAM83G localised primarily to the cytoplasm but also to the nucleus [3,57,66]. As reported previously, FAM83H showed both cytoplasmic and nuclear speckles [52,53,57]. Crucially, all FAM83 proteins co-localised with the CK1 isoforms they interacted with (Figure 5A) [57]. The CK1-isoform selective nature of FAM83 interaction was also evident in cells, as FAM83F only co-localised with CK1α but not with CK1ε [57], while FAM83H co-localised with both CK1α and CK1ε [57]. FAM83 mutants that are no longer able to interact with CK1 isoforms fail to co-localise with CK1 isoforms in specific subcellular compartments that the wild-type FAM83 proteins do [57]. The binding of FAM83 members to CK1 does not appear to alter the enzymatic activity of CK1 isoforms [37,57]. Although some FAM83 members are phosphorylated by CK1 isoforms *in vitro*, the constitutive and potent nature of their interaction with specific CK1 isoforms suggests a regulatory role rather than a substrate : enzyme relationship, which is often defined by a transient association. These findings imply that the DUF1669 domain of FAM83 proteins acts as an anchor for CK1α, α-like, δ and ε isoforms to direct them to specific subcellular compartments and, potentially, specific substrates, perhaps in much the same way as A-kinase-anchoring proteins (AKAPs) serve to localise protein kinase A to appropriate substrates [66]. Further regulation on subcellular localisation and affinity towards CK1 isoforms could be achieved through transcriptional or post-translational modifications of individual FAM83 proteins by different signalling cues. The organisation and streamlining of signal transduction by scaffolding and anchoring proteins has been reported for many cellular processes [67].

## The interaction between FAM83 proteins and CK1 isoforms is critical in cellular processes and signalling

CK1 isoforms are known to play critical roles in the Wnt/β-catenin signalling pathway [61]. They phosphorylate many components of the Wnt/β-catenin signalling pathway, including β-catenin, and exert both negative and positive roles in Wnt/β-catenin signalling [61,68–73]. This suggests that CK1 activity in Wnt signalling is carefully regulated, but how this is co-ordinated to allow execution of these opposing outcomes in Wnt/β-catenin signalling remains elusive. We discovered that PAWS1-mediated activation of Wnt/β-catenin signalling necessitated its association with CK1α [37]. PAWS1 mutations incapable of binding CK1 fail to activate Wnt signalling and to induce Wnt-dependent axis duplication in *Xenopus* embryos [37]. Studies on HCT116 colorectal cancer cells and ameloblast cells also showed that the interaction between FAM83H and CK1α was essential for correct organisation of the keratin cytoskeleton, as a mutant of FAM83H unable to interact with CK1α and chemical inhibition of CK1α hindered the proper organisation of the keratin cytoskeleton [49,52]. It has been proposed that many FAM83H mutations reported in AI, which result in truncated proteins that still have an intact DUF1669 domain, potentially cause the pathogenesis of the disease through mislocalisation of interacting CK1 isoforms [51]. In light of these data, it is increasingly evident that the many biological roles of FAM83 members are likely to be mediated through their association with specific CK1 isoforms (Figure 5B).

## Concluding remarks and future perspectives

The identification of the DUF1669 domain, which is conserved in FAM83 proteins, as an anchor for CK1 isoforms represents a significant development in attempting to establish the shared biochemical roles of the poorly characterised FAM83 family of proteins [57]. In the process, FAM83 proteins have emerged to be key regulators of CK1 isoforms that potentially explain the myriad of cellular processes that CK1 isoforms control (Figures 4 and 5). Whether the DUF1669 domain also harbours any pseudo-PLD functions, such as binding to specific phospholipids or other ligands, is still unclear. Indeed, if the pseudo-PLD domain did mediate interaction with phospholipids, it would be very interesting to establish whether such events would affect its ability to interact with the CK1 isoforms. Future structural insights of the DUF1669 domain in isolation and complex with CK1 isoforms will shed light into these possibilities, as well as the determinants of CK1-isoform selective interactions displayed by different FAM83 members. While the PAWS1 : CK1α and FAM83H : CK1α interactions have been shown to be crucial in mediating Wnt signalling [37] and keratin organisation [49,52], respectively, the key CK1α substrates that are affected by these FAM83 proteins remain to be defined. Therefore, precisely how FAM83 members regulate the function of CK1 isoforms in cells is still unclear. The next challenge in the field is to determine the CK1 substrate landscape that is determined by each individual FAM83 protein, within a specific signalling or physiological context. Furthermore, because FAM83 proteins interact robustly with different CK1 isoforms and localise them to different subcellular compartments [57], any physiological changes caused by overexpression of FAM83 proteins have to be considered as possible consequences of CK1 mislocalisation. In a similar vein, different physiological responses that have been attributed to specific CK1 isoforms can now be evaluated based on its association with a specific FAM83 member and its expression and subcellular distribution. It is unclear whether FAM83 proteins are the only regulators of CK1 isoforms in cells. Knocking out PAWS1 from U2OS cells resulted in depletion in endogenous CK1α protein levels, which could be rescued by reintroduction of WT PAWS1 but not by CK1 interaction deficient mutant of PAWS1 [37], suggesting that the stability of the CK1α pool bound to PAWS1 relies on the expression of PAWS1. The RNA helicase DDX3, which lacks the DUF1669 domain, has also been reported to interact with CK1ε, to regulate Wnt/β-catenin signalling [74]. Clearly more work needs to be done in order to establish the extent to which CK1 isoforms are controlled by FAM83 members, and whether some members act redundantly to control CK1 function. Although FAM83 members are only present in vertebrates, CK1 isoforms are also conserved in invertebrates. This suggests that the mode of CK1 regulation in invertebrates is distinct from vertebrates. Despite the involvement of CK1 isoforms in many diseases, their pleiotropic nature has earned them the label of ‘undruggable’ targets [63]. The emergence of the FAM83 proteins as key regulators of different CK1 isoforms provides new chinks in the armour for potential therapeutic opportunities to target specific CK1 isoforms in different physiological contexts.

## Abbreviations

AI: Amelogenesis imperfecta
BMP: bone morphogenetic protein
CK1: casein kinase 1
DYNLL1: dynein light chain 1
EGFR: epidermal growth factor receptor
FAM83: FAMily with sequence similarity 83
HMECs: human mammary epithelial cells
MAPK: mitogen activated protein kinase
PLDc: PLD catalytic
PLD: phospholipase D
